# Lip Epidermoid Cyst Caused by a Piercing: A Report of a Rare Case

**DOI:** 10.1155/2022/8015990

**Published:** 2022-02-08

**Authors:** Takashi Ito, Satoshi Fukuzawa, Kenji Yamagata, Shohei Takaoka, Makiko Ohkubo-Sato, Fumihiko Uchida, Naomi Ishibashi-Kanno, Hiroki Bukawa

**Affiliations:** Department of Oral and Maxillofacial Surgery, Faculty of Medicine, University of Tsukuba, Tennodai 1-1-1, Tsukuba, Ibaraki 305-8575, Japan

## Abstract

We report the case of a lip epidermoid cyst, caused by piercing in a 23-year-old Japanese woman. She had an exophytic lesion in the lower lip associated with the piercing which was initially diagnosed as a mucous retention cyst. The lesion was resected under local anesthesia, and pathological examination revealed an epidermoid cyst, likely caused by piercings. Piercing-induced epidermoid cysts frequently occur in the tragus. There have been no reports of piercing-induced epidermoid cysts developing in the oral cavity. To our knowledge, this is the first report of a lip epidermal cyst caused by piercings. Six months have passed since the operation, and it has not recurred.

## 1. Introduction

Body piercings have emerged as a component of recent fashion trends. They are worn on the ears and oral region, specifically the tongue and lips [1]. Epidermoid cysts are encountered in all parts of the body and occur 1.6% of times in the oral cavity [2]. There are also rare reports that it has become malignant [3]. However, to our knowledge, lip epidermal cysts caused by piercings have not been reported. We report the case of an epidermoid lip cyst caused by piercings.

## 2. Case Report

The patient was a 23-year-old Japanese woman with unremarkable medical and family histories. Five years prior, she had her lower lip pierced, such that it penetrated the left side of the lip and the skin. She stopped wearing her piercings after a few months, and the piercing holes closed spontaneously. Wound indurated or swelling was not noted.

When she was 22 years old, she had her left lower lip pierced again. The earrings were inserted twice. However, the pierced hole from the first set of piercings had been occluded. A mass appeared on the lower lip two months later, and the patient reported to our department due to its persistence.

**Figure 1:**
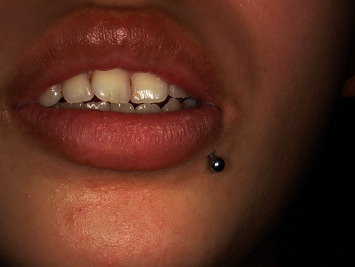
Extraoral findings.

**Figure 2:**
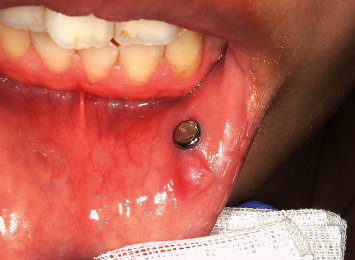
Intraoral finding. Tumor formation on the lip mucosa on the mesial side of the lip piercing.

Intraoral examination revealed a palpable mass, measuring approximately 4 × 4 mm, on the left lower lip. The piercing was in contact with the mass and penetrated the skin and mucosa of the lower-left lip (Figures 1 and 2).

**Figure 3:**
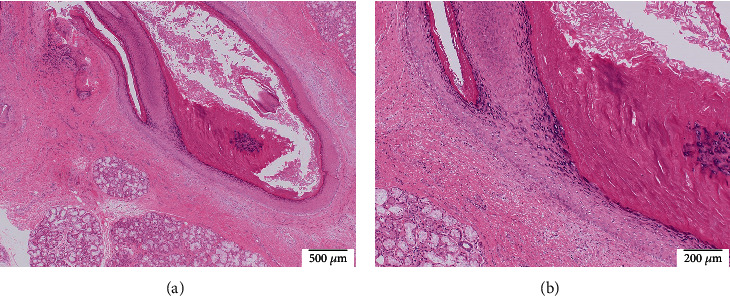
Histopathological findings of the lip cyst. (a) Hematoxylin and eosin stain ×4. (b) Hematoxylin and eosin stain ×100. A cyst covered with keratinized stratified squamous epithelium. Keratin is stored in the cyst.

She was clinically diagnosed with a mucous retention cyst of the lower lip. She was instructed to stop using her piercings two weeks before undergoing the lesion resection. The pierced hole was closed intraoperatively. The lesion and the scar on the pierced hole were resected under local anesthesia. Histopathological examination revealed a keratin-containing cyst covered with keratinized stratified squamous epithelium. Therefore, the patient was pathologically diagnosed with an epidermoid cyst (Figure 3).

**Figure 4:**
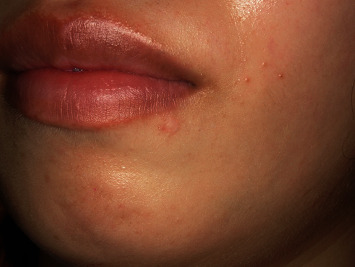
Extraoral finding 10 months after the surgery.

**Figure 5:**
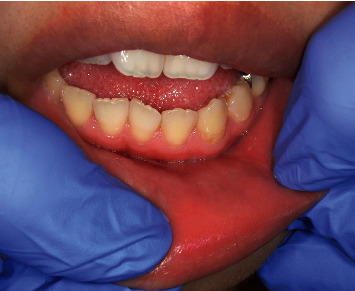
Intraoral finding 10 months after the surgery. The wound 10 months after surgery. No recurrence was noted.

One year has passed since the operation, and the wound has resolved without infection or recurrence (Figures 4 and 5).

## 3. Discussion

Piercings has been often applied to the tragus. Piercing complications in the tragus reportedly occur in 35% [4]. Piercings have main complications including infections, allergic reactions, keloid formation, and traumatic lacerations.

Epidermal cysts are soft tissue cysts that commonly develop in the head and neck regions. Congenital causes are associated with invasion and residual embryonic ectoderm. These causes acquired epithelial tissue invasion, such as trauma and inflammation [5].

Kim et al. reported that 14 out of 132 people with tragus piercing complications have experienced epidermoid cysts [6]. Epidermoid cysts, caused by piercings, occur during infection or bleeding. Epidermoid cysts reportedly develop when patients stop using their piercings [7].

Epidermoid cysts are attributed to epithelial invasion and remnants at the ectodermic fusion in the congenital and to the implantation of keratinized epithelium by trauma in the acquired cases [5, 8]. Epidermoid cysts on the lip due to trauma have also been reported due to denture irritation [9].

Chen et al. were the first to report the complications of oral piercings in 1992, and several cases have been reported after that [10]. The major complications of oral piercing are gingival recession and tooth fractures [11–13]. However, epidermoid cysts have not been reported, as complications associated with lip piercings. To the extent of our knowledge, this was the first case report on the occurrence of lip epidermoid cysts with such causality. In our case, the piercings were inserted twice. However, the pierced hole from the first set of piercings had occluded. The epidermoid cyst formation possibly occurred during that time. Alternatively, the epithelium might have invaded under the lip membrane after the second insertion, forming an epidermoid cyst. According to the patient, the cyst developed two months after she had gotten her second set of piercing. However, she did not notice the cyst during the second insertion. Based on this, the second insertion likely caused epithelial invagination.

The process of receiving piercings is invasive, but the publicity materials on its complications have been few.

Since piercings have emerged as a component of the recent fashion trends, cases like the present case may become more common in the future. Thus, dentists and oral surgeons should consider this a differential diagnosis. Moreover, people should be informed regarding the potential complications and risks associated with oral piercings.

## Data Availability

The data used to support the findings of this study are included within the article.
